# RETRACTED: Kaim et al. Why Does Monk Fruit Extract Remain Only Partially Approved in the EU? Regulatory Barriers and Policy Implications for Food Innovation. *Foods* 2025, *14*, 2810

**DOI:** 10.3390/foods15183303

**Published:** 2026-09-18

**Authors:** Urszula Kaim, Urszula Gawlik, Katarzyna Lisiecka

**Affiliations:** 1 Department of Bioprocess Engineering, Wrocław University of Economics & Business, St. Komandorska 118-120, 53-345 Wrocław, Poland; 2 Department of Biochemistry and Food Chemistry, University of Life Sciences in Lublin, St. Akademicka 13, 20-950 Lublin, Poland; urszula.gawlik@up.lublin.pl (U.G.); katarzyna.lisiecka@up.lublin.pl (K.L.)

The journal retracts the article titled “Why Does Monk Fruit Extract Remain Only Partially Approved in the EU? Regulatory Barriers and Policy Implications for Food Innovation” [1], cited above.

Following publication, concerns were brought to the attention of the Editorial Office regarding the accuracy of the cited sources in this publication [1].

In accordance with the journal’s standard procedures, the Editorial Office and Editorial Board conducted an investigation that identified a significant number of non-existent references in this article [1], which were determined to undermine the overall reliability of this study. Although the authors cooperated with the investigation and submitted a revised version, the explanations and supporting materials provided were not deemed sufficient by the Editorial Board to resolve the identified concerns.

As a result, the Editorial Board has decided to retract this publication as per MDPI’s retraction policy.

This retraction was approved by the Editor-in-Chief of the journal *Foods*.

The authors have been informed of this retraction and have indicated their agreement with this decision.
